# Task-irrelevant stimuli robustly drive phasic pupil-linked arousal but only weakly affect memory formation

**DOI:** 10.1038/s41598-026-65835-4

**Published:** 2026-08-17

**Authors:** J. Hebisch, P. Van Puyenbroeck, L. Schwabe, J. W. de Gee, T. H. Donner

**Affiliations:** 1 Section Computational Cognitive Neuroscience, Department of Neurophysiology and Pathophysiology, University Medical Center Hamburg-Eppendorf, Hamburg, Germany; 2 Zurich Center for Neuroeconomics (ZNE), University of Zurich, Zurich, Switzerland; 3 University Research Priority Program Adaptive Brain Network Mechanisms in Development and Learning (URPP AdaBD), University of Zurich, Zurich, Switzerland; 4 Department of Cognitive Psychology, Institute of Psychology, University of Hamburg, Hamburg, Germany; 5 Cognitive and Systems Neuroscience, Swammerdam Institute for Life Sciences, University of Amsterdam, Amsterdam, Netherlands; 6 Amsterdam Brain & Cognition, University of Amsterdam, Amsterdam, Netherlands; 7 Bernstein Center for Computational Neuroscience, Charité Universitätsmedizin, Berlin, Germany

**Keywords:** Neuroscience, Psychology, Psychology

## Abstract

Brainstem arousal systems including the locus coeruleus noradrenaline system, respond transiently to behaviorally relevant events. This also drives dilations of the pupil, which are often observed during cognitive tasks. The strength of pupil responses during encoding of stimulus material predicts the success of its later retrieval, which might reflect the impact of noradrenaline on synaptic plasticity and memory formation. The pupil also dilates in response to task-irrelevant sounds, which could therefore serve as a valuable tool for investigating causal effects of phasic, pupil-linked arousal on cognition. Here, we evaluated whether task-irrelevant white noise sounds affect memory formation and memory-based decisions. These sounds were played before, during or after the presentation of memoranda (images or spoken words). Memory success was measured in recognition and free recall tasks the day after. Trial-to-trial variations in the amplitude of pupil dilations during word encoding in the absence of task-irrelevant sounds predicted memory success. Task-irrelevant white-noise sounds also robustly dilated the pupil but did not yield strong evidence of improved memory success: later recall of words was unaffected, and recall of images was weakly boosted only when the sounds were presented concurrently with them. Our results suggest that pupil-linked arousal processes triggered by task-irrelevant sounds differ from those recruited endogenously during memory formation.

**Supplementary Information:**

The online version contains supplementary material available at 10.1038/s41598-026-65835-4.

## Introduction

Not all memories are remembered with equal ease. Prioritizing the encoding and consolidation of some memories over others enables the brain to store only what will likely be relevant in the future^1–3^. For example, memories encoded under high emotionality are generally retrieved better^1,4^. Mounting evidence suggests that the prioritization of memory formation by behavioral relevance may be mediated by the arousal systems of the brainstem^5–7^, in particular the locus coeruleus (LC) noradrenaline (NA) system. With its widely distributed projections to the forebrain and impact on its target circuits, the LC-NA system is in an ideal position to control network states^8^ as well as synaptic plasticity in the cerebral cortex^9–11^ and hippocampus^12–14^, which is, in turn, required for memory formation^15–17^.

The brain’s arousal systems, including the LC-NA system, activate transiently during cognitive tasks^18–21^, which also drives dilations of the pupil^22,23^. Thus, pupil responses under conditions of constant luminance have often been used as a marker of transient (“phasic”) arousal responses^24^. Emotional stimuli evoke a larger pupil response during encoding than neutral stimuli^4,25,26^. The amplitude of these pupil responses, in turn, predicts subsequent memory success^4,27–29^. While such results are consistent with a role of brainstem arousal systems in memory formation, these findings are correlational in nature.

Task-irrelevant sounds have emerged as a promising non-invasive method for causally driving pupil^22,30–34^ as well as LC responses^22,35^. Because biases in perceptual decisions are affected by optogenetic LC stimulation in mice^19^ and pharmacological catecholamine boost in humans^36^, we previously tested the effects of task-irrelevant sounds on perceptual decision-making^31^. While the task-irrelevant sounds evoked reliable pupil dilations, they did not affect biases or any other aspect of perceptual decision-making^31^. These findings cast doubt on the suitability of task-irrelevant sounds as a tool for effectively modulating decision-making, but do not necessarily imply a failure to trigger activity in the brainstem arousal system. For example, it is possible that white noise stimuli drive responses in arousal circuits or LC sub-populations that do not affect perceptual decision-making^19^ but may still impact other types of cognitive processes.

In the present study, we tested the possibility that task-irrelevant sounds boost memory encoding success. In contrast to the domain of perceptual decision-making, in which arousal is considered to influence short-term neural processing, in the domain of memory, manipulations of phasic arousal may affect cognitive performance not by modulation of cortical activity but by modulation of plasticity. Specifically, given the established role of neuromodulation in synaptic plasticity^9,10,37^, a memory-enhancing effect of a phasic boost of pupil-linked arousal is plausible and would offer valuable insights into the function and specificity of brainstem arousal systems. To this end, we asked participants to encode either spoken words or images in memory, followed by free recall and a recognition memory test on the next day. Task-irrelevant white noise sounds were played at different time points during encoding as well as the recognition task. If task-irrelevant sounds effectively drive LC responses, they should dilate the pupil and, critically, boost memory success for the images or words that are paired with the sounds during encoding. The (“old” versus “new”) recognition task enabled us to test for an effect of task-irrelevant white noise sounds on memory decision bias, which, just as perceptual decision bias^21,31,38^, negatively correlates with the amplitude of task-evoked pupil responses^39^.

## Results

**Fig. 1:**
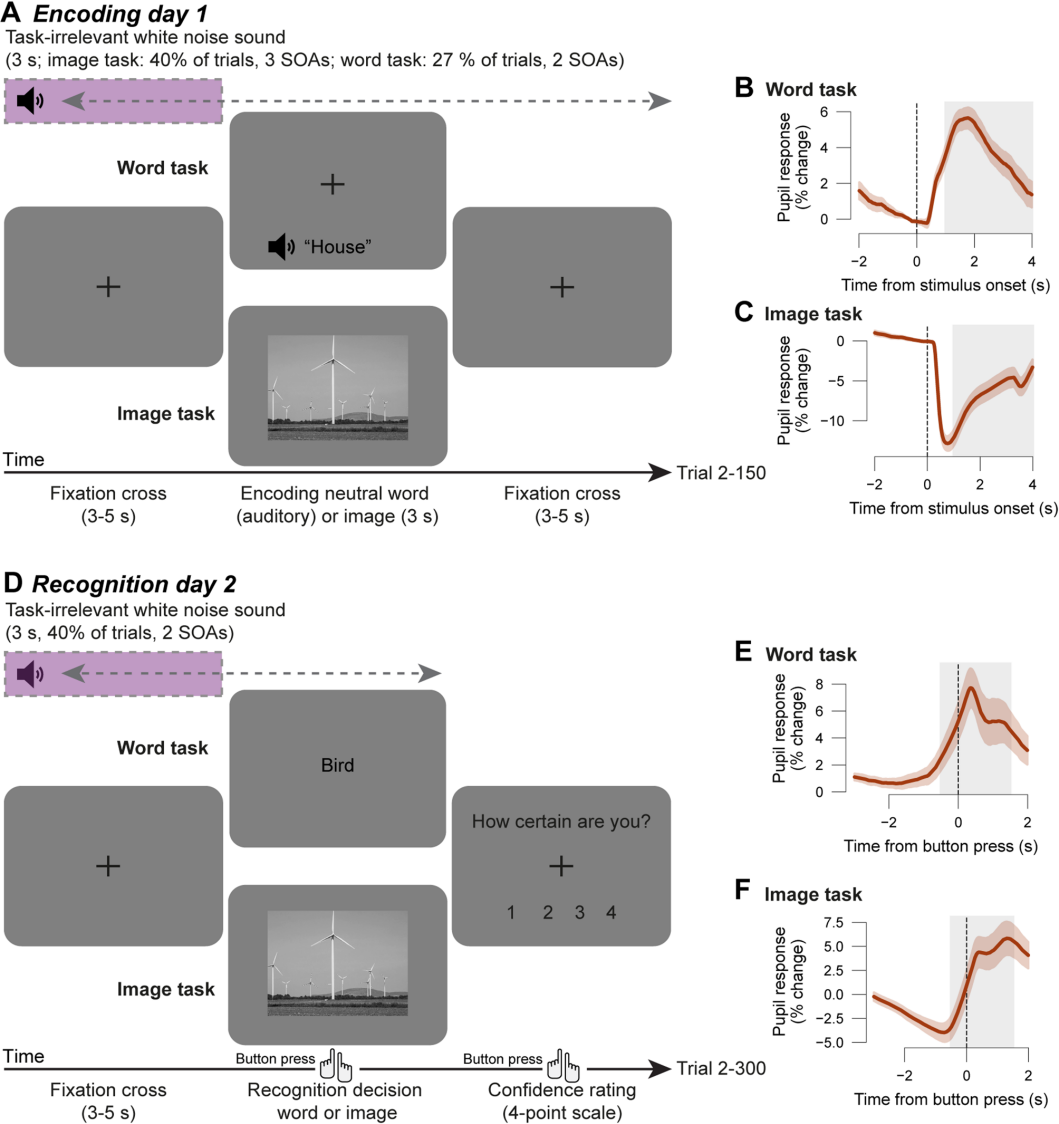
Behavioral tasks and pupillometry. **(A)** Schematic sequency of trial events during encoding (day 1). The word task comprised 60 words (presented via headphones), and the image task comprised 150 greyscale images (mean luminance matched with grey background). The task-irrelevant, auditory white noise sound appeared before or after word presentation on a total of 27% of trials, and before, during, or after image presentation on a total 40% of trials. **(B)** Average pupil response time course during encoding, locked to word onset. Lines, group average; orange shading, SEM across participants; grey shading, time window used for quantifying task-evoked pupil responses. **(C)** Same as (B) but for the image task. **(D)** The recognition task (day 2) consisted of 120 visually presented words or 300 images respectively and task-irrelevant sounds were played before or during stimulus presentation on a total of 40% of trials. **(E, F)** As (B-C) but for pupil responses during recognition task, locked to choice (button-press).

Participants intentionally encoded 60 neutral words and 150 greyscale neutral images on day 1 of the experiment (Fig. 1A; see “Methods”). Task-irrelevant sounds were played on 27% (word task) or 40% (image task) of trials with an onset time before, during (only image task) or after stimulus presentation (randomly selected). Task-irrelevant sounds consisted of 3 s of white noise at 75 dB, which evoked robust pupil dilations in our previous work^31^. Right after encoding, participants were asked to freely verbally recall as many stimuli as they could. Day 2 of the experiment (24 h later) started with another free verbal recall of the stimuli, followed by a recognition task (Fig. 1D). In the latter, we presented 120 words and 300 images, half of which were repeats from day 1 and half of which were new. Participants were asked to report whether they thought the stimulus was “old” or “new” by pushing one of two buttons. They then had to indicate their confidence in the decision on a scale from 1 to 4. During the recognition task, task-irrelevant sounds were again played on 40% of randomly selected trials, either before or during stimulus presentation.

The mean task-evoked pupil responses on trials without any task-irrelevant sounds replicated effects previously reported for the encoding task^4^: pupil dilation locked to word presentation (Fig. 1B) and constriction locked to image presentation (Fig. 1C). In the recognition tasks for both stimulus domains, we found pupil dilations around the time of the button press (Fig. 1E, F), likely reflecting phasic task engagement^21,39,40^.

### Evoked pupil responses during word encoding predict memory success

A previous study found a correlation between the amplitude of pupil responses during stimulus encoding and subsequent memory performance^4^. Such an effect was observed for both emotional words and images, but when stimuli were neutral, it was only observed for words, not for images^4^. We replicated the effects for neutral stimuli in our current dataset, which did not contain emotional stimuli.

**Fig. 2:**
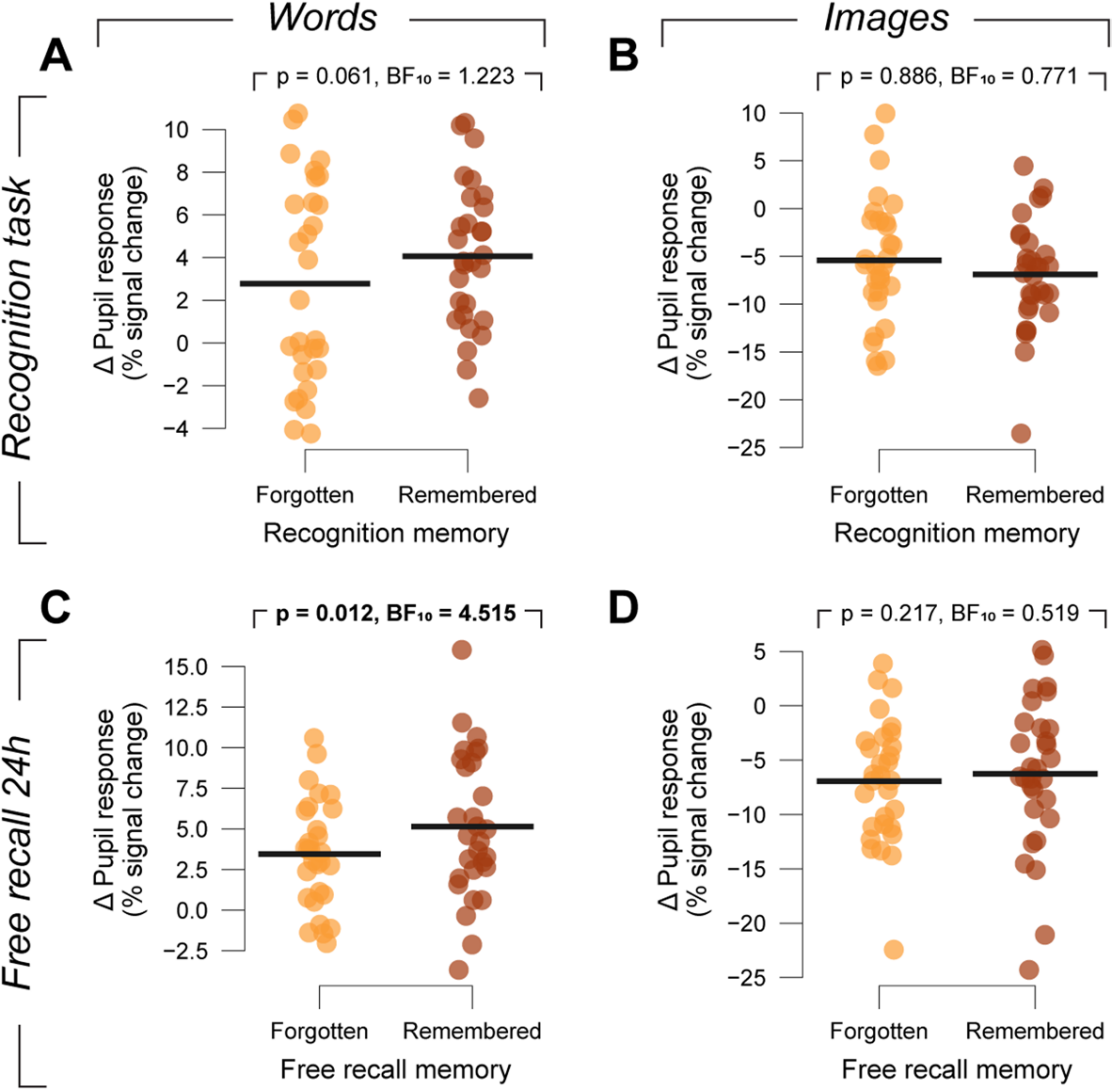
Amplitude of pupil responses during encoding sorted by subsequent memory performance. **(A)** Individual trial-average pupil response amplitudes during encoding (collapsed across an interval of 1–4 s from word onset) on trials with no task-irrelevant sounds. Trials are sorted by whether words were recognized (remembered) or missed (forgotten) in the recognition task on day 2. Black bars, group average. **(B)** As (A), but for images. **(C**,** D)** As (A) and (B), but for words and images that were recalled vs. not recalled 24 h later.

We quantified pupil response amplitude as the mean response over a window of 1–4 s following stimulus onset on trials without task-irrelevant sounds (see “Methods”) to capture the full extent of task-evoked (i.e. likely stimulus encoding-evoked) variability in pupil size (Fig. 1B, C). Words that were remembered in free recall (day 2) elicited a larger pupil response during encoding than words that were later forgotten (t = 2.402, *p* = 0.012, one-sided t-test; BF_10_=4.515; Fig. 2C). Splitting pupil responses based on whether the stimuli were successfully remembered in the recognition task yielded a similar effect, which was only trending (t = 1.596, *p* = 0.061, one-sided t-test; BF_10_=1.223; Fig. 2A; see Fig. S2 for immediate free recall). However, no effects related to memory success were evident for the image task (BF_10_ ranging from 0.519 to 0.771; Fig. 2B, D; pupil response time courses split by memory success, Fig. S1). In sum, we found a weak, but significant relationship between pupil responses during encoding of neutral words and later memory success that was qualitatively similar to previously reported results^4^.

### Task-irrelevant sounds drive pupil dilations

**Fig. 3:**
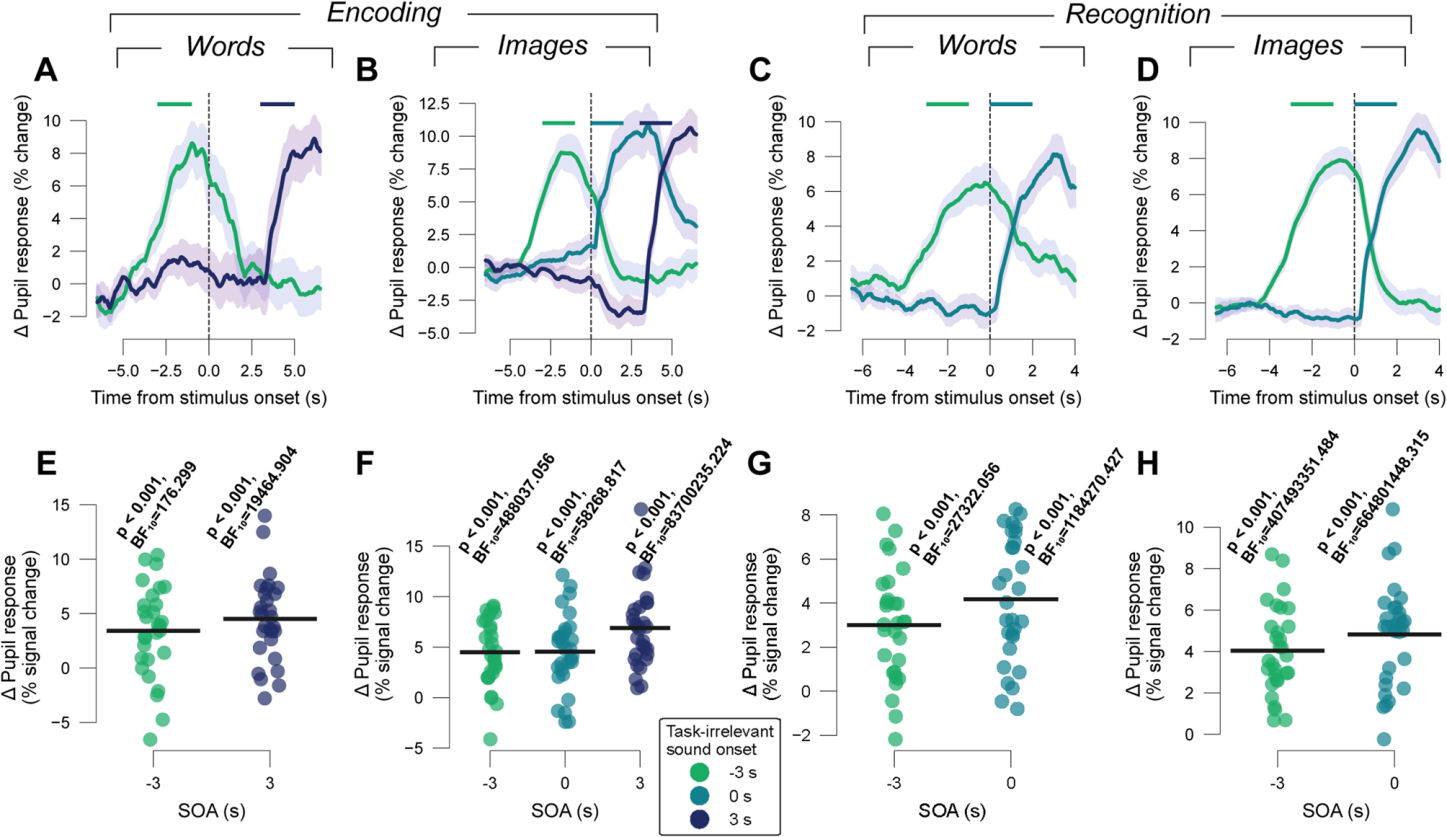
Robust pupil responses to task-irrelevant sounds. **(A)** Differential pupil response time courses between trials with and without task-irrelevant sounds in the word encoding task. Time courses were baseline-corrected with mean value from interval from 3.5 to 3 s prior to stimulus onset. Shading, SEM across participants. Top bars, intervals used for quantifying task-irrelevant sound-evoked pupil responses for the respective sound onsets. **(B)** As (A) but for image encoding task. **(C, D)** As (A-B), but for the recognition tasks. **(E)** Individual amplitudes of sound-evoked pupil responses in the word encoding task. Black bars, group average. P and BF_10_, p-values and Bayes factors (two-sided t-tests against zero). **(F)** As (E) but for the image encoding task. **(G, H)** As (E-F), but for the recognition tasks.

We next isolated the pupil response evoked by the task-irrelevant sound by computing differential response time courses between trials with and without task-irrelevant sounds (see “Methods”). For each stimulus onset asynchrony (SOA) between task-irrelevant sound and memorandum onset, the differential time course showed clear dilations for roughly 4 s (i.e. until 1 s after the sound terminated, Fig. 3A-D), peaking at about 10% signal change.

We quantified the amplitude of the sound-evoked pupil response by averaging the differential response time courses across 2 s following sound onset. Most participants exhibited reliable positive (i.e., dilatory) effects (Fig. 3E-H), with highly significant group-level effects of task-irrelevant sounds for each task (main effects of sound condition: word encoding task: F_(2,56)_ = 15.849, *p* < 0.001; image encoding task: F_(3,87)_ = 34.93, *p* < 0.001; word recognition task: F_(2,56)_ = 31.975, *p* < 0.001; image recognition task: F_(2,58)_ = 71.507, *p* < 0.001; repeated measures ANOVAs). This was further supported by Bayesian t-tests for the different SOAs (see “Methods”; range of BF_10_: from 176.299 to 664801448.315). These results replicate our previous research that comprehensively quantified effects of task-irrelevant white noise sounds of different durations (including the 3 s used here) on pupil responses in the context of a perceptual decision task^31^.

### Inconsistent evidence for boost of memory formation by task-irrelevant sounds

**Fig. 4:**
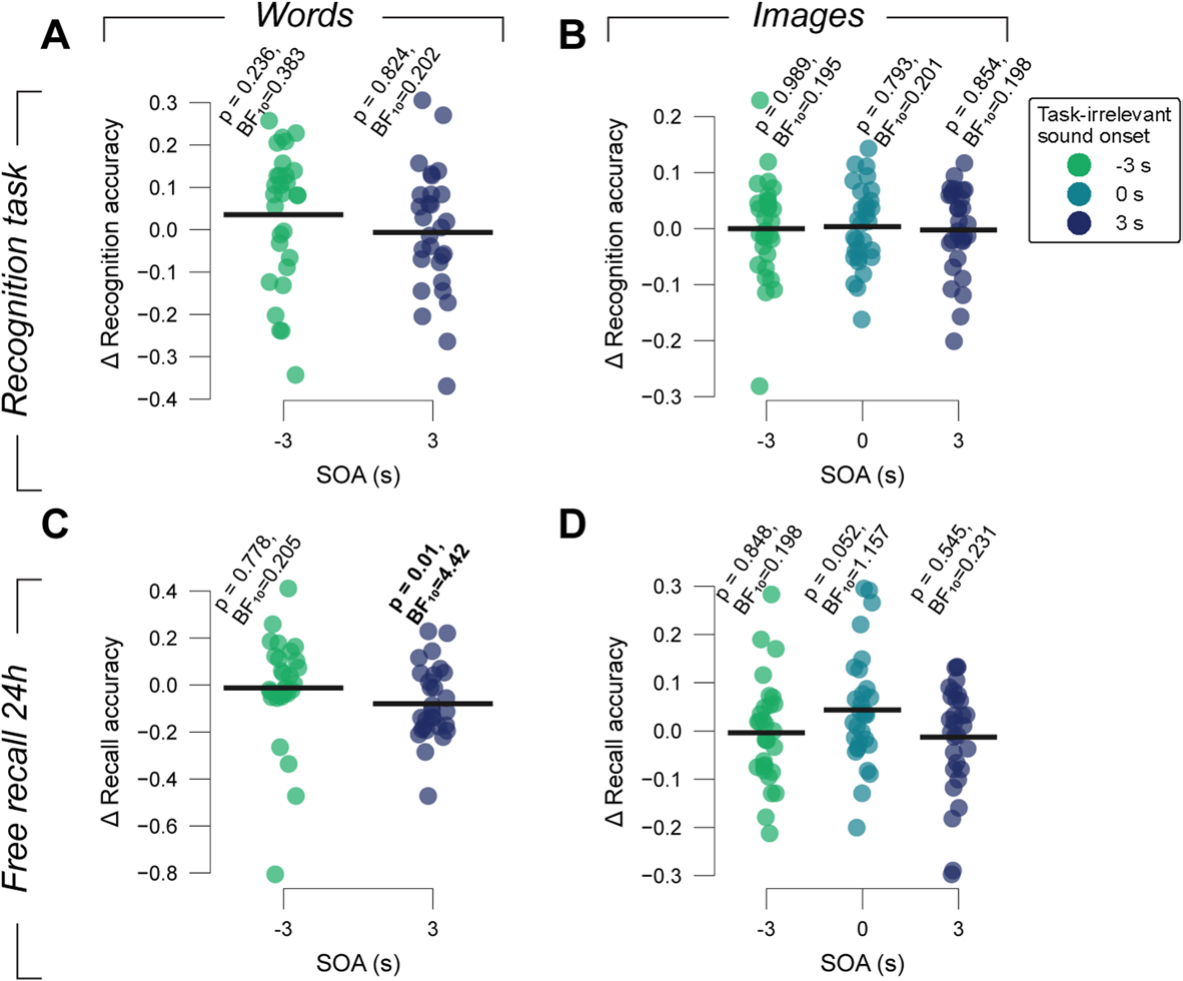
Difference in memory performance due to task-irrelevant sounds in encoding. **(A)** Difference of recognition accuracy between trials with and without task-irrelevant sounds (played in the word encoding task) for each individual, sorted by sound SOA. Black bars, group average. P and BF_10_, p-values and Bayes factors for t-tests against zero. **(B)** As (A), but for the image task. **(C-D)** As (A-B), but for accuracy during free recall on day 2.

Despite robustly driving pupil dilations, the task-irrelevant sounds had no consistent, detectable effect of boosting memory formation (Fig. 4). In the word task, we observed no evidence for a main effect of sound condition (i.e., no sound, sound before or after stimulus presentation in the encoding task) on the success of recognition (Fig. 4A: F_(2,56)_ = 1.022, *p* = 0.366) or free recall (Fig. 4C: F_(2,56)_ = 2.481, *p* = 0.093; see Fig. S2 for immediate free recall on day 1). The differences in recognition accuracy between trials with and without task-irrelevant sounds were not likely different from zero for any SOA (BF_10_ range from 0.202 to 0.383). The same was true for the effect of the sound at the early onset asynchrony on memory accuracy on the free recall that took place right before the recognition task (BF_10_ = 0.205). However, words which were followed by the task-irrelevant sound were recalled significantly less often than those without task-irrelevant sounds during encoding (t = -2.748, *p* = 0.01, BF_10_ = 4.42). This may result from an interfering effect of the sounds after word presentation on memory encoding.

In the image task, the recognition performance also did not exhibit any effect of sound condition (Fig. 4B; F_(3,87)_ = 0.042, *p* = 0.988; SOA − 3 s: BF_10_ = 0.195, SOA 0 s: BF_10_ = 0.201, SOA 3 s: BF_10_ = 0.198). In the free recall on day 2, there was a trending main effect of sound condition (Fig. 4D: F_(3,87)_ = 2.248, *p* = 0.088). While sounds played during or after image presentation resulted in no difference in memory success (SOA − 3 s: t = -0.193, *p* = 0.848, BF_10_ = 0.198; SOA 3 s: t = -0.612, *p* = 0.545, BF_10_ = 0.231), the sounds played during image presentation had a trending, positive effect on recall performance (t = 2.025, *p* = 0.052, BF_10_ = 1.157). Notably, while the main effect was also only trending, the effect of sounds during image presentation was significant for the immediate free recall success on day 1 (Fig. S2D; F_(2,58)_ = 2.619, *p* = 0.056; SOA − 3 s: t=-0.206, *p* = 0.838, BF_10_ = 0.198; SOA 0 s: t = 2.308, *p* = 0.028, BF_10_ = 1.885; SOA 3 s: t = -0.026, *p* = 0.979, BF_10_ = 0.194).

The sound condition did not impact confidence ratings in the recognition tasks (word task: F_(2,56)_ = 0.077, *p* = 0.926, SOA − 3 s: BF_10_=0.209, SOA 3 s: BF_10_ = 0.214; image task: F_(3,87)_ = 0.065, *p* = 0.978, SOA − 3 s: BF_10_=0.208, SOA 0 s: BF_10_=0.195, SOA 3 s: BF_10_ = 0.199). In sum, we observed a mixed pattern of effects of task-irrelevant sounds during encoding on memory formation, with weak, not long-lasting evidence for a memory-boosting effect, specifically in the image (not words) task when sounds were presented concurrently with the images.

### Task-irrelevant sounds do not affect recognition decision

Recognition task performance does not only depend on the quality of memory encoding, but additionally on the (memory-based) decision process involved in deciding whether an image is old (i.e. familiar) or new (unfamiliar)^41^. Previous work established a negative relationship between the amplitude of pupil dilations during recognition decisions and the (commonly conservative, favoring “new” responses) bias in those decisions^39^. We replicated the above effect for the trials without task-irrelevant sound during the recognition task (Fig. S3). The task-evoked pupil responses around choice report varied substantially from trial to trial, with dilations on the most trials, but even robust constrictions from baseline on some others (Fig. S3A-B). The amplitude of the evoked pupil response correlated negatively with signal detection-theoretic measure of bias – decision criterion c – in the recognition decision: the larger the pupil response, the more negative c, so the larger the tendency to report that a stimulus was “old” (Fig. S3C-D, word task: group average correlation coefficient r_s_ = -0.196, t = -2.587, p = 0.015; image task: group average correlation coefficient r_s_ = -0.279, t = -4.317, p < 0.001). While there was a relationship of task-evoked pupil responses with bias, the pupil responses exhibited no relation (linear or polynomial, see “Methods”) to recognition sensitivity (signal detection-theoretic index d’; Fig. S3E-F). These results illustrate a distinct (monotonic) effect of phasic arousal responses on decision bias, in line with previous work c^39^.

**Fig. 5:**
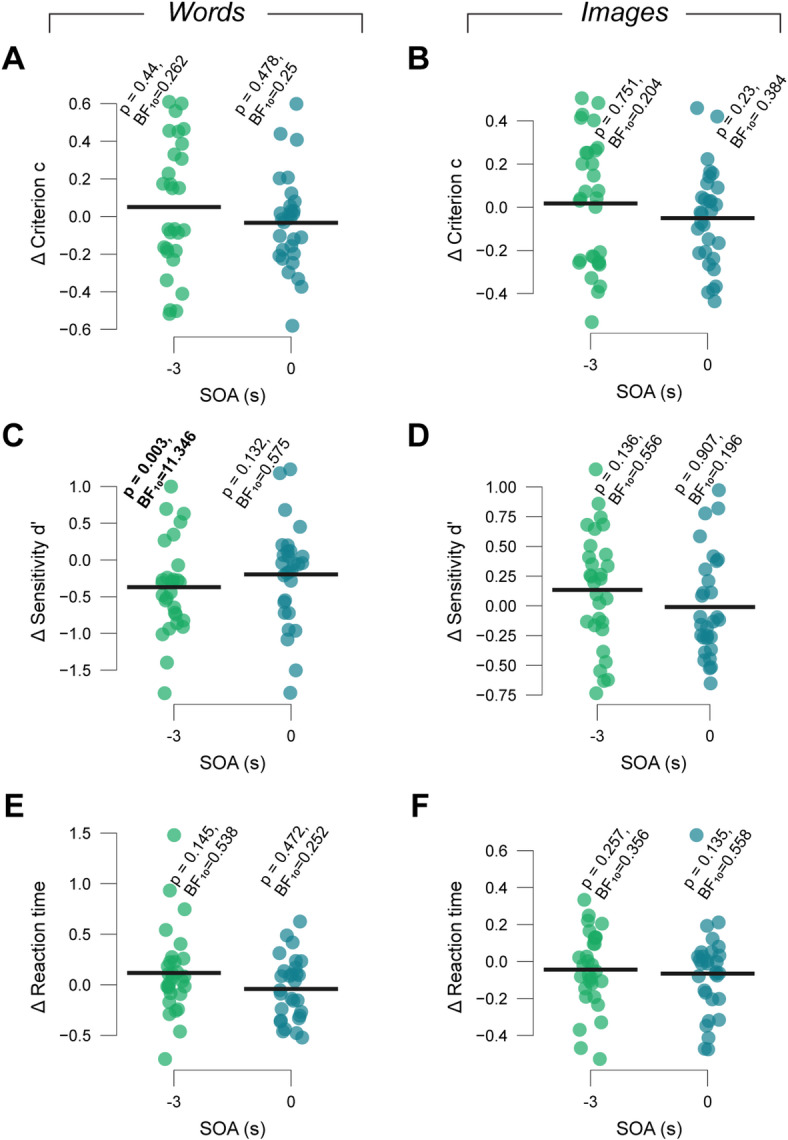
Behavioral effects of task-irrelevant sounds during recognition tasks. **(A)** Participant-wise difference of recognition decision bias (criterion c) on task-irrelevant sound trials versus trials with no task-irrelevant sound for the respective sound onset asynchrony with respect to word onset. Black bars, group average. P and BF_10_, p-values and Bayes factors for t-tests against zero. **(B)** As (A), but for the image recognition task. **(C-D)** As (A-B) but for recognition performance (sensitivity d’). **(E-F)** As (A-B) and (C-D), but for reaction time.

Despite these correlations between task-evoked pupil responses and decision bias (Fig. S3) as well as the robust pupil responses to task-irrelevant sounds during the recognition task (Fig. 3C-D, G-H), task-irrelevant sound conditions per se had no effect on recognition behavior (Fig. 5). We found no evidence for a modulation of decision bias (c) through task-irrelevant sounds in either the image or the word recognition tasks (word task: F_(2,56)_ = 0.859, p = 0.497, SOA − 3 s: BF_10_ = 0.262, SOA 0 s: BF_10_ = 0.25; image task: F_(2,58)_ = 0.847, p = 0.434, SOA − 3 s: BF_10_ = 0.204, SOA 0 s: BF_10_ = 0.384; Fig. 5A-B). The same was true for sensitivity (d’) in the image task (image recognition task: F_(2,58)_ = 1.649, *p* = 0.201; BF_10_ range from 0.196 to 0.556, Fig. 5C-D). Solely in the word recognition task, there was an effect of task-irrelevant sound (word recognition task: F_(2,56)_ = 3.724, *p* = 0.03, repeated measures ANOVA), where task-irrelevant sounds played before word presentation reduced sensitivity (t = -3.196, *p* = 0.003, BF_10_ = 11.346, Fig. 5C), again in line with distracting effect of the sounds. We also found no effect of task-irrelevant sounds on reaction times (word recognition task: F_(2,56)_ = 2.901, *p* = 0.063, SOA − 3 s: BF_10_=0.538, SOA 0 s: BF_10_ = 0.252; image recognition task: F_(2,58)_ = 1.115, *p* = 0.335, SOA − 3 s: BF_10_ = 0.356, SOA 0 s: BF_10_ = 0.558; Fig. 5E-F) or confidence ratings (word recognition task: F_(2,56)_ = 1.956, *p* = 0.151, SOA − 3 s: BF_10_=1.887, SOA 0 s: BF_10_ = 0.279; image recognition task: F_(2,58)_ = 0.396, *p* = 0.675, SOA − 3 s: BF_10_ = 0.291, SOA 0 s: BF_10_ = 0.243).

## Discussion

Previous correlational studies have pointed to a key role for phasic, pupil-linked arousal in the prioritization of some memories over others^4–6,27–29,42^, in line with a role of neuromodulators – including NA – controlling central arousal states, in cortical plasticity. Here, we tested if such prioritization of memories of words or images could be experimentally controlled by driving phasic arousal via task-irrelevant sounds. We found that words that were later remembered tended to be associated with larger pupil dilations during encoding compared to later forgotten words. Task-irrelevant sounds played during encoding evoked robust pupil responses in both tasks and at all latencies, but only weakly improved memory recall on the same day, and only for the image task and when presented concomitantly with the images. In contrast, we found a negative effect on the free recall on the next day for words that were followed by a task-irrelevant sound. For all other sound onset times, there was no evidence for an effect of the sounds on word or image memory recall. Furthermore, accuracy in the word and image recognition tasks was not impacted by the sounds during encoding at all. It is unlikely that this inconsistency of behavioral effects of task-irrelevant sounds on memory performance can be attributed to the strength of the manipulation: the magnitude of pupil dilations evoked by the sounds exceeded the difference in pupil response between remembered and forgotten words (compare Figs. 2 and 3A-D). In other words, the predictive value of pupil dilation per se for memory success found in previous work^4^ and replicated here, does not hold for all contexts.

Loud sounds, such as the ones we used here, drive LC activity and dilate the pupil^22,35,43^. Such an activation of the LC-NA system as well as other neuromodulatory systems controlling arousal would be a plausible mechanism, by which task-irrelevant sounds might boost memory formation. There is strong evidence that phasic LC activation gates synaptic plasticity in the hippocampus^12^ and cerebral cortex^9–11^, which is in turn critical for memory formation^16,17^. For example, NA release shapes long-term potentiation and depression in the hippocampus^13,14,44^. The positive effect on the immediate free recall that we found for sounds played during image presentation may indeed reflect such a neuromodulation-related boost of memory formation. If so, this boosting mechanism would be temporally specific because the (weak) effect in the image task was not evident when sounds occurred 3 s before or after images, but only when co-occurring with the images in a 3 s time window. That would also explain why we did not find the effect in the words task, where we omitted that SOA (i.e., no sounds played during the presentation of words). Alternatively, images accompanied by sounds may have boosted sensory encoding of the images through phasic arousal, resulting in improved memory.

The memory-boosting effect of sounds during image presentation on their later recall was, however, weak and inconsistent. For example, there was no effect in the old-new recognition task or for SOAs other than 0 s. Furthermore, we found the opposite (negative) effect of sounds played after the presentation of words, which impaired recall success the day after. This negative effect might result from the task-irrelevant sounds interfering with the encoding of previously heard words. Indeed, previous work shows that interference shortly after encoding may play a central role in forgetting^45^. The fact that we did not observe such a negative effect for sounds played 3 s after the image encoding may be due to the difference in modality between the task-irrelevant stimulus and the stimulus to be encoded, or the superposition of memory-boosting and processing-interference effects, or a combination of both.

The neural control of pupil-linked arousal is complex, entailing the LC as well as other brainstem centers, such as the cholinergic forebrain^46^, the serotonergic dorsal Raphe nucleus^23^, or superior and inferior colliculi^22^. At a functional level, pupil-linked arousal is related to a multitude of cognitive processes, such as attention allocation, mental effort or uncertainty^30,47–49^. Pupil-linked arousal during attention allocation may facilitate the processing of relevant information^30^, while increases in arousal due to uncertainty may impact belief updating^30,48,50,51^. Differences in these processes could potentially lead to the distinct (lack of) behavioral correlates of endogenous, task-evoked pupil-linked arousal and externally sound-evoked pupil-linked arousal.

Against this backdrop, the observed combination of robust pupil dilations without concomitant memory improvement under task-irrelevant sounds that we found for most of the sound onset times may also reflect insufficient activity in the arousal systems of the brainstem accompanied by increased activity in other nuclei in the pathway responsible for dilating the pupil. In fact, loud sounds evoke even larger responses in the inferior colliculi than in the LC^22^, which are part of the early auditory pathway^52^, sufficient to dilate the pupil^22^, but play no obvious role in memory formation. Finally, it is possible that the sounds did drive LC neurons, but a different subpopulation of those neurons than the ones required for memory formation, for example due to different patterns of projections to the forebrain^19,53,54^.

Previous work reported a lack of behavioral effects of sound manipulations across different cognitive domains^31,32^. In the context of perceptual decision-making, we previously found this lack of effects in a rigorous test of the sound manipulation in terms of duration, intensity and timing^31^. In the context of memory, one other study found no behavioral effect of different types of task-irrelevant sounds on face recognition performance while showing similar pupil dilations we report here^32^. However, as the authors of this study concluded, the lack of effect on recognition performance could have been due to misaligned timing: in their experiment, sounds were played exclusively before stimulus onset. The systematic manipulation of sound onset times relative to the memoranda allows us to get a more complete picture here. In addition, we extended that previous work by testing memory 24 h after encoding instead of in the same session only, which (i) established an effect of task-evoked pupil dilation on long-term memory success and (ii) ruled out that overnight consolidation may culminate in a beneficial impact of task-irrelevant sounds on memory. Together, the findings highlight the importance of future studies testing for generalization of the timing-specific memory-boosting effect we found for natural visual images to other modalities (e.g. spoken words). Direct recordings or fMRI of the responses of brainstem nuclei involved in the control of pupil size (such as LC) concurrently with our task and sound manipulation will help pinpoint the neural basis of the effect we observed.

We also studied the effects of the task-irrelevant sounds during the recognition task as well as of trial-to-trial variations in task-evoked pupil responses. Regarding the former, we found no clear impact of task-irrelevant sounds on decision behavior, in line with findings from the domain of perceptual decision-making^31^. Regarding the latter, we found a negative, linear relationship between recognition task-evoked pupil response magnitude and decision bias (i.e. old/new tendency) in both the word and the image task. This effect may reflect an influence of arousal on the weighting of priors relative to incoming evidence in the decision process^18,21,55,56^, whereby the evidence is here sampled from memory^57^.

The ability to experimentally (and non-invasively) manipulate phasic arousal in a manner that mimics the physiological, phasic recruitment of arousal systems during cognitive tasks is an important goal for current neuroscience. Such an ability could profoundly advance the mechanistic understanding of the effect of phasic neuromodulation on plasticity, learning, and memory. Our results highlight (i) the challenges faced by such approaches due to the heterogeneity underlying pupil-linked, phasic arousal responses and (ii) the importance of the precise timing of such manipulations relative to the cognitive task at hand. The result suggests that task-irrelevant sounds are not sufficient to reliably drive arousal transients necessary for robust and persistent effects on memory-based behavior. In sum, there remains an urgent need for research on manipulation approaches for phasic arousal.

## Methods

### Participants

33 healthy volunteers (19 female, 14 male; age range, 20–33 y) participated in this experiment. We calculated an a priori sample size using G*power and effect sizes reported in Bergt et al.^4^. Based on the magnitudes of the effects of stimulus emotionality on pupil response (images: d = 2.15; words: d = 0.56) and of remembering (versus forgetting) on pupil size (images: n_p_^2^ = 0.09, negative images only: d = 0.76; words: n_p_^2^ = 0.15), for one-sided paired sample t-tests and repeated measures ANOVA with a power of 0.8, this revealed required sample sizes between 4 and 22 participants.

All participants had normal or corrected-to-normal eyesight without wearing glasses. Participants were instructed not to drink any beverages containing any caffeine up to two hours prior to the sessions.

All participants gave written informed consent and were remunerated by the hour (15 €/h). The experiment was conducted in accordance with the Declaration of Helsinki. It was approved by the ethics committee of the Faculty of Psychology and Human Movement Sciences at the University of Hamburg. Participants were excluded post-hoc if they showed a low number of valid trials (< 250) after removing trials with more than 20% missing pupil data (see analysis of pupil responses) or reaction times below 200 ms or longer than 8 s. This resulted in a sample size of 30 participants for the image tasks and 29 participants in the words task.

### Procedure

The experiment consisted of two consecutive sessions that took place on subsequent days, with a delay of about 24 h. Each session lasted approximately 2 h. In both sessions, participants were asked to sit at a desk resting their head on a chin rest at 50 cm distance of the VIEWPixx monitor (1920 by 1080 pixels) with a refresh rate of 100 Hz. Luminance levels were kept stable throughout the whole task. The experiment took place in a dark room (0 lx). The screen color was kept stable throughout the experiment tasks at an average RGB value of [128, 128, 128]. The first session consisted of an image encoding task, followed by an immediate free recall, and then a word encoding task, followed by a free recall as well. The second session consisted of: the free recall of images, the free recall of words, an image recognition task, and finally a word recognition task. This experimental design was adapted from the one used by Bergt et al.^4^. Stimulus sets contained neutral images and words (written and audio). These included stimuli used by Bergt et al.^4^ and images from the THINGS database^58^. Images were presented in greyscale and were modified using the Pillow toolbox in python^59^ to have the equal average luminance, which was the same as the grey background.

### Apparatus

The experiment was controlled by a personal computer using the MATLAB Psychophysics Toolbox^60^. Auditory stimuli were played using AKG K72 headphones.

The gaze direction and pupil size of participants’ right eyes were tracked using an Eyelink 1000 (SR Research, Osgoode, Ontario, Canada). The eye data was sampled at a rate of 1000 Hz and an average spatial resolution of 15 to 30 min arc. At the beginning of each task, the eye tracker was calibrated. Obtaining data from one eye is standard practice as there is no evidence pointing to differences in pupil responses between both eyes in participants without neurological disorders^61^.

### Encoding tasks

Trials in the image and word encoding tasks consisted of a pre-stimulus interval (3–5 s), a stimulus presentation interval (3 s) and a post-stimulus interval (3–5 s). During the pre- and post-stimulus intervals, only a fixation cross was shown on the grey background. In the image encoding task, we presented an image overlaid with a fixation cross during the full duration of the stimulus presentation interval. In total, participants saw 150 images. In the word encoding task, participants heard a word in the beginning of the stimulus presentation interval (average duration ~ 1 s) while only a fixation cross was presented on the screen. In total, participants heard 60 words. They were instructed to intentionally encode the stimuli and knew that they would be tested on them in the second session of the experiment.

On a fraction of trials, a task-irrelevant sound (auditory white noise, 75 dB, 3 s duration) occurred on one of multiple possible stimulus onset asynchronies (SOAs). These trials were randomly selected under the constraint that the same number of sounds are played at each SOA. In the image encoding task, this task-irrelevant sound was played on 40% of trials with an onset either in the pre-stimulus interval (3 s prior to image onset), at the same time as image onset or in the post-stimulus interval (3 s after image onset). In the word encoding task, it was played on 27% of trials either in the pre- or the post-stimulus interval.

### Free recall tasks

In the free recall task, participants were asked to verbally recall as many stimuli as possible. For the image recall task, this meant that participants first mentioned as many details about each image as they could describe with ease. If the experimenter was unsure which image was described back to them, they asked to describe the image in more detail until they could either identify the image, or the participant could not go into more detail. During this task, participants sat behind a curtain to prevent effects of non-verbal communication between participants and experimenter.

### Recognition tasks

In the recognition tasks, each trial consisted of a pre-stimulus interval (3–5 s), a decision interval (terminated by button press), and a confidence rating interval (terminated by button press). The pre-stimulus intervals consisted only of a fixation cross shown on the grey background. During the decision interval, a stimulus was presented visually and participants were requested to indicate whether this stimulus had been presented on the day before or not by button press on a computer keyboard (button “1” for old stimuli and “0” for new ones). In the confidence rating interval, participants were asked to indicate how certain they were of their answer on a scale from 1 (“not certain”) to 4 (“very certain”). In a randomized order, 300 images (150 old) were presented in the image recognition task, and 120 words (60 old) were presented in the word recognition task.

The task-irrelevant sounds (same duration and intensity as during encoding) were played on 40% of trials in the recognition tasks. They occurred either in the pre-stimulus interval (SOA − 3 s with regard to stimulus onset) or started at the same time as the stimulus presentation in the decision interval. Trials were assigned randomly under the constraint that each SOA occurred the same number of times and that “old” stimuli paired with a sound in the recognition tasks were equally likely to have been paired with a sound in the encoding task compared to stimuli not paired with a sound in the recognition task.

### Analysis of pupil responses

Pupil data were analyzed using Python with a similar pipeline as in previous studies^21,31^.

#### Preprocessing

We used the Eyelink software for blink and saccade detection and a custom algorithm for detecting additional blinks missed by the software^62^. Missing data (mainly due to blinks) was linearly interpolated between 200 ms before and 200 ms after the missing data. We corrected for pupil responses to blinks and saccades with the help of a double gamma function convolution^63^. Finally, the cleaned time series was converted from arbitrary size units provided by the eye tracker (based on pixel number) to percent modulation around the median value.

#### Quantification of task-evoked pupil responses

For the encoding tasks, we quantified evoked pupil responses locked to the stimulus onset (i.e. image or word onset, respectively) on trials with no task-irrelevant sound. Epochs with more than 20% of missing data were excluded from analysis. The remaining epochs were then baseline-corrected by subtracting the mean pupil size over a window of 0.5 s prior to stimulus onset. The trial-average amplitude values of these task-evoked pupil responses were computed by averaging pupil response values across the interval from 1 s to 4 s after stimulus onset. This window was chosen in accordance with previous studies^4,40^ to account for the delay and sluggishness of the pupil response^40,64^. However, the results of the corresponding analysis remained unchanged if a time window from 1 s to 3 s after stimulus onset was applied (i.e., until the end of the image presentation; the duration of the words was variable). We used linear regression to remove variations in pupil response due to pre-trial baseline pupil size. The resulting amplitudes of task-evoked pupil responses during encoding were sorted according to whether the stimuli of the respective trials were subsequently remembered or forgotten, in either the free recall or the recognition tasks on day 2 (Fig. 2).

For the recognition tasks, we used epochs locked to the choice report (button press) on trials without task-irrelevant sounds. Again, epochs with more than 20% of missing data were excluded from the analysis, epochs were baseline-corrected by subtracting the mean pupil size over a window 0.5 s before stimulus onset, and the trial-average amplitude values of these task-evoked pupil responses were calculated by averaging pupil response values in the interval − 0.5 s to 1.5 s from button-press, again chosen based on previous work quantifying decision-related pupil responses^31,40^. We used linear regression to remove variations in pupil response due to pre-trial baseline pupil size or reaction time. The resulting amplitudes of task-evoked pupil responses during recognition were sorted into eight bins, within each participant (Fig. S3A-B).

#### Quantification of task-irrelevant sound-evoked pupil responses

To quantify the pupil responses evoked by the task-irrelevant sounds, we baseline-corrected epochs locked to the task-relevant stimulus onset (subtracting the mean over 0.5 s before stimulus onset), for both the encoding and the recognition tasks. Epochs with more than 20% of missing data were excluded from analysis. We then calculated the mean pupil response time courses on trials with no task-irrelevant sound and subtracted this from each pupil response time course on single trials containing a task-irrelevant sound. The resulting differential pupil epochs of trials with task-irrelevant sound were then realigned to sound onset and again baselined with the mean over a 0.5 s interval before sound onset, yielding the final sound-evoked response time courses, which were binned by SOA and then averaged. The trial-average sound-evoked pupil response amplitudes were finally quantified as the mean differential pupil size over a window of 2 s following sound onset.

### Analysis of choice behavior

For all behavioral analyses, we excluded the first 10 trials of the respective task of interest due to potential effects of strong decrease in baseline pupil size in the beginning of each experimental block (compare Hebisch et al.^31^.

For analysis of effects of the task-irrelevant sound during encoding on memory performance, we computed the mean memory accuracy on immediate free recall, 24 h free recall and recognition task as the fraction of correctly recalled or recognized stimuli of all stimuli presented on day 1 for the image and the word task separately. This was done separately for trials with and without task-irrelevant sounds, the former sorting by SOA of the task-irrelevant sound.

For relating trial-to-trial variations of pupil responses or the task-irrelevant sounds during recognition to behavioral performance, we computed the signal detection-theoretic indices sensitivity d’ and criterion c^65,66^ as well as reaction time for each respective task-evoked pupil response bin or task-irrelevant sound SOA. The sensitivity reflects one’s ability to discriminate old stimuli from new stimuli. It is calculated as the difference between z-scored hit rates and false alarm rates. The criterion describes the systematic bias to classify a stimulus as old (“liberal” bias) or new (“conservative” bias). It is calculated as the average of z-scored hit and false alarm rates multiplied by -1.

### Statistical comparisons

We compared task-evoked pupil responses during encoding between stimuli that were later remembered and those that were forgotten during the free recall tasks or the recognition task using paired sample t-tests and by determining the corresponding Bayes factors (Fig. 2).

For assessing the effect of task-irrelevant sounds on pupil size and behavioral metrics, we applied repeated measures ANOVAs with the (no-) task-irrelevant sound condition (i.e. no sound vs. individual SOAs) either during encoding or during recognition as the within-subject (4-level) factor. We further scrutinized the relationships testing the differences in the variable of interest between the no-task-irrelevant sound condition and the respective SOA condition against zero by means of Bayesian t-tests (Figs. 3, 4 and 5).

We tested the relationships between task-evoked pupil responses during the recognition task and recognition behavior (Fig. S3). For each participant, we computed the Spearman (rank order) correlation between each of the behavioral metrics and the bin-wise task-evoked pupil amplitudes. We also ran a second-order polynomial regression using the bin-wise average task-evoked pupil response amplitude as predictor of the behavioral metrics. The sample of correlation coefficients and regression weights was then compared against 0 with a paired-samples t-test.

## Supplementary Information

Below is the link to the electronic supplementary material.

Supplementary Material 1

## Data Availability

The datasets generated and analyzed during the current study can be found online at https://doi.org/10.25592/uhhfdm.18985. Analysis scripts are available under https://github.com/DonnerLab/2026_Hebisch_task-irrelevant-sounds-boost-pupil-only-weak-effect-on-memory.
